# Polypoid Dysplasia in Barrett's Esophagus: Diagnosis, Management, and Very Different Outcomes in Two Consecutive Cases

**DOI:** 10.1155/2016/8421531

**Published:** 2016-11-23

**Authors:** Megan Murphy, Christina Tofani, Kunjal Gandhi, Anthony Infantolino

**Affiliations:** ^1^Department of Internal Medicine, Thomas Jefferson University Hospital, Philadelphia, PA 19107, USA; ^2^Department of Gastroenterology & Hepatology, Thomas Jefferson University Hospital, Philadelphia, PA 19107, USA

## Abstract

*Background*. Barrett's esophagus is associated with an increased risk of adenocarcinoma. Dysplasia in Barrett's esophagus is a precursor to adenocarcinoma. Rarely, dysplastic polypoid lesions are superimposed on Barrett's esophagus. Most reported cases of polypoid dysplasia in Barrett's esophagus have been advanced on presentation and treated with esophagectomy. We describe two cases of polypoid changes in Barrett's esophagus and treatment with polypectomy followed by radiofrequency ablation.* Cases*. A 75 yo male presented with esophageal polyps, which on biopsy showed gastric cardia/foveolar mucosa with focal intestinal metaplasia without dysplasia. Biopsy of intervening flat mucosa was consistent with nondysplastic Barrett's esophagus. Extensive hot snare polypectomies were performed followed by RFA. One year later, repeat EGD revealed no evidence of Barrett's esophagus. A 61 yo male presented with esophageal polyps, which on biopsy showed gastric cardia/foveolar mucosa with intestinal metaplasia and foci of low-grade dysplasia. Extensive hot snare polypectomies were performed followed by RFA. At repeat EGD, four months later, an esophageal mass was found. Biopsy of the mass showed invasive adenocarcinoma. The patient was referred for esophagectomy.* Conclusion*. This case series shows two outcomes, one with successful eradication of dysplasia and the other with disease progression to invasive adenocarcinoma requiring esophagectomy.

## 1. Introduction

Barrett's esophagus with dysplasia rarely presents as several polypoid esophageal lesions. To date, the management of this presentation is not standardized. Herein, we report two cases of polypoid dysplastic Barrett's esophagus managed with hot snare polypectomies followed by radiofrequency ablation.

### 1.1. Patient 1

**Figure 1:**
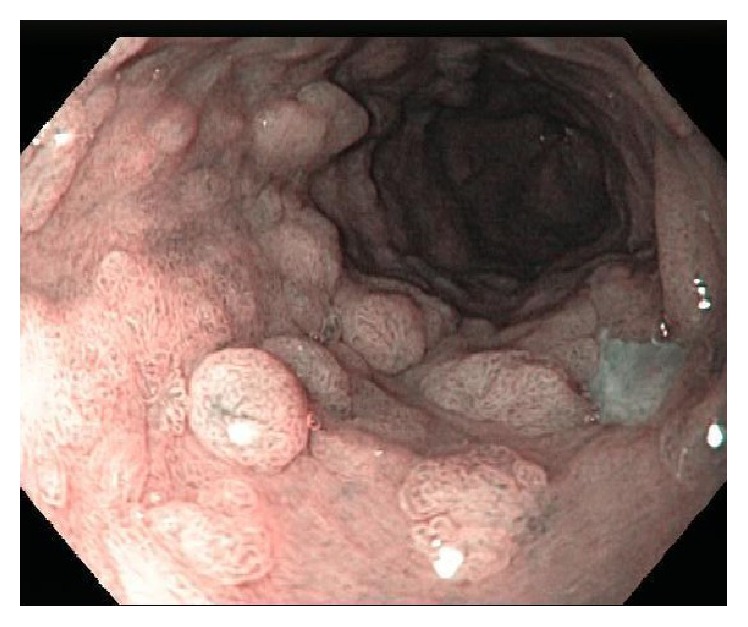
Patient 1. Original EGD (NBI). Several short-stalked polypoid lesions in the esophagus.

**Figure 2:**
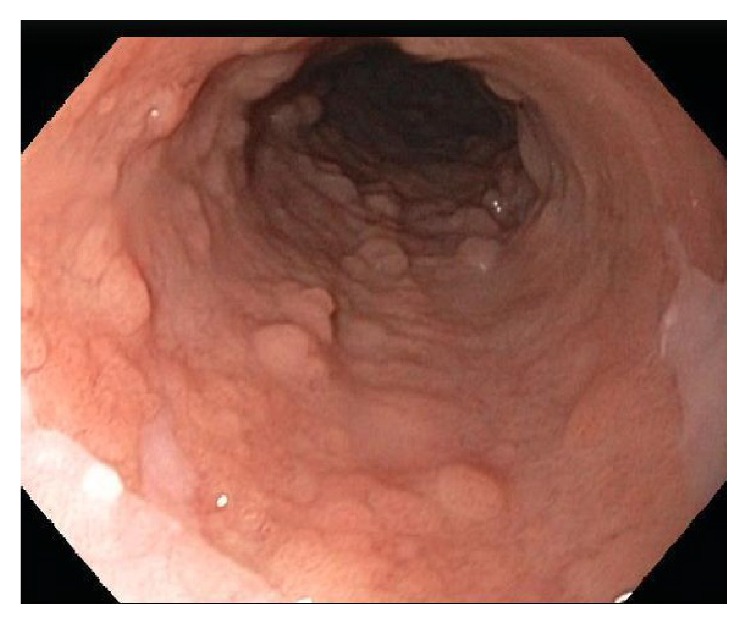
Patient 1 (white light endoscopy). Esophagus after multiple hot snare polypectomies, prior to RFA.

**Figure 3:**
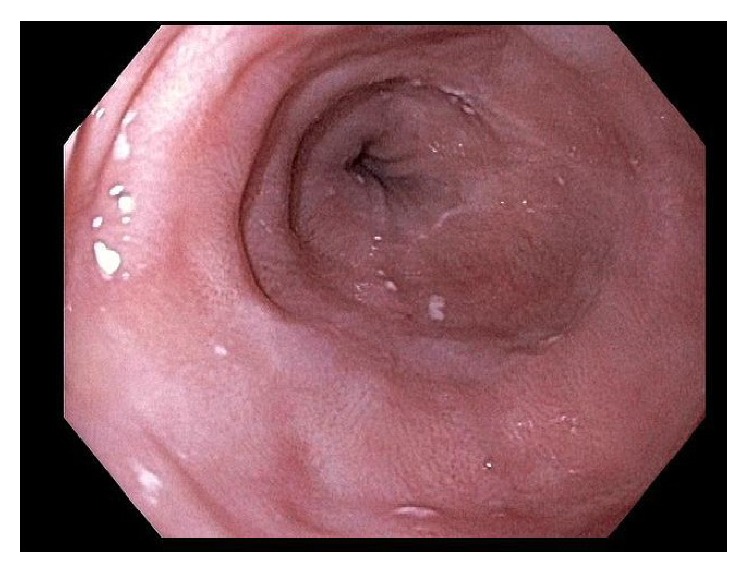
Patient 1 (white light endoscopy). Esophagus with squamous-appearing mucosa after RFA.

A 75-year-old male with history of chronic gastroesophageal reflux disease presented with a known history of esophageal polyps. The patient was not felt to be a good surgical candidate due to multiple comorbid medical conditions and the patient was not interested in the surgical option. An esophagogastroduodenoscopy (EGD) (Figure 1) and endoscopic ultrasound (EUS) were performed in October 2013. Barrett's esophagus was noted at 31–40 cm (C9M9) from the incisors along with multiple frond-like, short-stalked polypoid lesions, which were removed by multiple hot snare polypectomies. EUS showed wall layers were intact with no surrounding adenopathy. All pathology specimens were reviewed by an expert gastrointestinal pathologist. Pathology from polypectomies showed gastric cardiac/foveolar type polyps with focal intestinal metaplasia and reactive glands and was negative for dysplasia. Biopsies of the surrounding flat mucosa were consistent with Barrett's esophagus, glandular epithelium with focal metaplasia and negative for dysplasia. The patient returned in January 2014 for repeat EGD. Again, multiple frond-like, short-stalked polypoid lesions were superimposed on Barrett's esophagus (Figure 2). Multiple hot snare polypectomies were performed and sent for histologic evaluation. The pathology at this time showed low-grade dysplasia with focal high-grade dysplasia arising in extensive intestinal metaplasia. Given the extensive nature of the disease and high perioperative risk secondary to medical comorbidities, the patient was not deemed a candidate for endoscopic submucosal dissection (ESD) or esophagectomy. The patient returned two months later, in March 2014, for repeat EGD and radiofrequency ablation (RFA) therapy. Subsequent RFA procedures were performed at 3, 6, and 9 months. Pathology from June 2014 showed the esophageal nodule to be consistent with hyperplastic gastric polyp formation and no evidence of adenomatous changes. In June 2015, one year after the last RFA treatment, EGD with esophageal biopsies showed Barrett's esophagus and benign squamous mucosa without pathologic change (Figure 3). No polyps were visualized at that time. A volumetric laser endomicroscopy (Ninepoint®) procedure was also performed and it failed to reveal any evidence of subsquamous Barrett's esophagus. The patient was instructed to follow up for continued close surveillance.

### 1.2. Patient 2

**Figure 4:**
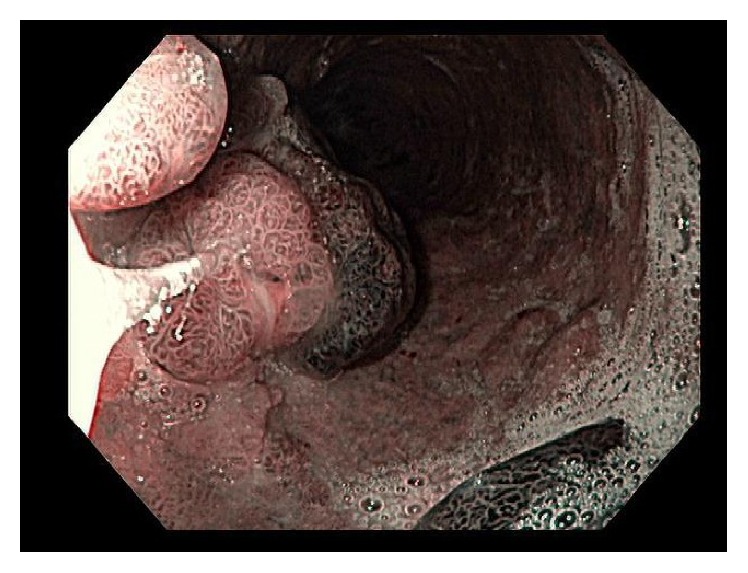
Patient 2. Original EGD (NBI). Several short-stalked polypoid lesions in the esophagus.

**Figure 5:**
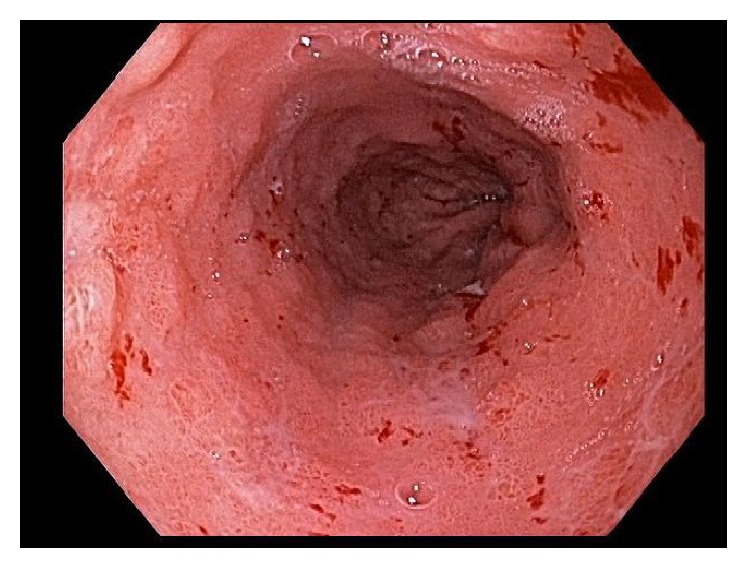
Patient 2 (white light endoscopy). Esophagus after multiple hot snare polypectomies and RFA.

**Figure 6:**
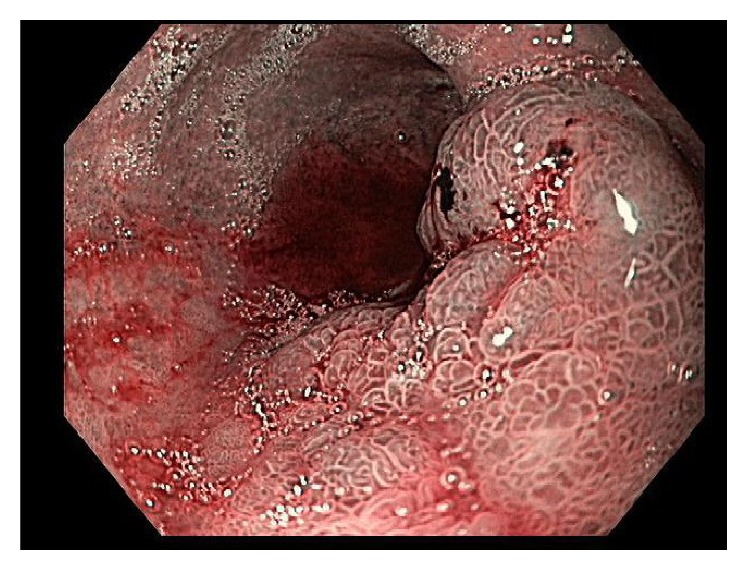
Patient 2 (NBI). New esophageal mass.

A 61-year-old man with known long segment Barrett's esophagus and esophageal polyps presented for evaluation. An EGD (Figure 4) and EUS were performed January 2010. Barrett's esophagus was noted at 20–40 cm (C20M20) from the incisors. Two nodules were noted at 22 and 24 cm and were removed by endomucosal resection (EMR). EUS showed nodules to be confined to the mucosa, possible submucosa, and no lymph nodes were identified. All pathology specimens were reviewed by an expert gastrointestinal pathologist. Pathology for both nodules was determined to be inflamed glandular mucosa with intestinal metaplasia negative for dysplasia. Due to the uncertain nature and the extent of the polypoid Barrett's esophagus, after several discussions, the patient was offered an evaluation for esophagectomy but refused at that time. Due to the 20 cm segment length, extensive ESD was not considered an option. Repeat EGD in March 2015 showed a segment from 20 to 40 cm compatible with Barrett's esophagus with multiple frond-like, short-stalked polyps scattered throughout. Many of the polyps were removed by snare cautery and the intervening mucosa was biopsied. Surrounding flat mucosa was confirmed Barrett's esophagus with low-grade dysplasia. Pathology of the polyps showed foci of low-grade dysplasia, foveolar type, arising in gastric cardiac type mucosa with intestinal metaplasia. The patient returned two weeks later in April 2015 for further polypectomies. Ten semipedunculated polyps of multilobular appearance, ranging from 4 to 8 mm in size, in the middle and lower third of the esophagus were removed by snare cautery. Again, foci of low-grade dysplasia were identified on pathology. One month later, the patient underwent his first RFA treatment with no complications (Figure 5). Four months later in September 2015, repeat EGD was performed. An ulcerated, nonbleeding mass was visualized in the middle third of the esophagus at 31–33 cm (Figure 6). Biopsy showed invasive carcinoma with high and low-grade dysplasia. Given these findings, subsequent RFA treatments were cancelled and the patient was referred to surgery for esophagectomy evaluation.

## 2. Discussion

Barrett's esophagus is acquired metaplasia of the esophagus and is associated with increased risk of adenocarcinoma, presumably through a metaplasia-dysplasia-carcinoma sequence [1]. The overall prevalence of Barrett's esophagus is 1.6%, with the majority of patients not progressing beyond nondysplastic intestinal metaplasia or low-grade dysplasia [2, 3]. However, in those who advance to high-grade dysplasia, the risk of adenocarcinoma may be as high as 10% per patient-year [4]. Thus, it is important to identify and stratify any dysplastic changes. Typically, dysplasia in Barrett's esophagus occurs as flat or nodular areas. However, it rarely can occur as a stalked, polyp-like lesion. Studies on the few reported cases of polypoid dysplasia have shown that Barrett's esophagus-associated polypoid dysplasia shares similar clinical, pathological, and molecular features as flat dysplasia, including a predisposition to adenocarcinoma [5]. However, optimal management remains unclear.

Radiofrequency ablation (RFA) is a known treatment option that has the potential to eradicate dysplasia and metaplasia in a significant amount of patients with Barrett's esophagus, thus decreasing their risk for adenocarcinoma [6]. However, given the infrequency of polypoid dysplasia of Barrett's esophagus, it has not been reported if the same outcomes are to be expected.

A review of the literature yielded six case studies with polypoid dysplastic lesions within Barrett's esophagus. Five of the cases contained adenocarcinoma and were treated with esophagectomy [6–10]. In one case, piecemeal polypectomies were performed with biopsy forceps and pathology showed tubular adenomas with low-grade dysplasia. However, through close-interval endoscopic surveillance, a recurrence of the lesion with high-grade dysplasia was detected and subsequently treated with esophagectomy [11].

Case 1 presented above is the only reported case of a dysplastic polypoid lesion completely treated with hot snare polypectomy followed by RFA. The patient, as described in the case, did have eradication of the dysplastic pathology, consistent with treatment of flat dysplasia in Barrett's esophagus. Conversely, case 2 represents the aggressive nature of esophageal adenocarcinoma. After one treatment of RFA, the patient progressed from low-grade dysplastic changes to invasive adenocarcinoma over the course of four months. Similar to previously described cases, the histologic changes were too advanced for RFA therapy and esophagectomy was recommended. Both patients were appropriately surveyed during treatment with esophageal biopsies following the Seattle protocol [12]. Currently, a surgical approach to polypoid BE with dysplasia may be the most definitive way to manage this disease. However, as presented in our two cases, endoscopic therapy may be a viable treatment option for those patients that either refuse or are not candidates for surgery. We suggest that, if appropriate, a surgical option always be offered to the patient.

In summary, true polypoid dysplasia, unlike the more common nodular dysplasia, in Barrett's esophagus is quite rare, with very few cases in the literature. However, they appear to share similar clinicopathologic and molecular features as typical dysplasia seen in Barrett's esophagus. In the literature, all cases of polypoid were treated with esophagectomy when adenocarcinoma or high-grade dysplasia was identified. In case 1 presented above, the patient had eradication of low- and high-grade dysplastic tissue after treatment with RFA. However, case 2 had progression of disease to invasive carcinoma in the setting of one RFA treatment and esophagectomy was recommended. Further studies of dysplastic polypoid changes are warranted to determine the efficacy of RFA therapy in this setting and to elucidate the disease course of these lesions. However, in the setting of Barrett's esophagus with dysplastic polypoid lesions in nonsurgical candidates or those who refuse surgery, we suggest considering EMR/polypectomy to achieve flat mucosa and RFA therapy in concert with close endoscopic surveillance as a treatment option. At centers capable of ESD, ESD for less extensive Barrett's esophagus is also an option, although the complication risk may be higher.
